# The association between autism spectrum disorder and congenital malformations: a population-based nested case-control study

**DOI:** 10.1038/s41380-025-03302-8

**Published:** 2025-10-15

**Authors:** Rony Cleper, Ori Kapra, Nadav Goldental, Raz Gross

**Affiliations:** 1 Gray Faculty of Medical & Health Sciences, Tel Aviv University, Tel Aviv, Israel; 2 Department of Epidemiology and Preventive Medicine, Gray Faculty of Medical & Health Sciences, Tel Aviv University, Tel Aviv, Israel; 3 The Chaim Sheba Medical Center, Ramat Gan, Israel; 4 Department of Psychiatry, Gray Faculty of Medical & Health Sciences, Tel Aviv University, Tel Aviv, Israel

**Keywords:** Autism spectrum disorders, Predictive markers

## Abstract

We examined whether specific congenital malformations (CM) detected at birth are associated with increased likelihood of autism spectrum disorder (ASD), by a case-control study nested within a 12-year birth cohort derived from the Israel National Birth Registry. The cohort included all registered ASD cases (n = 2099) and 1:1 age- and sex- matched controls. Overall, CM were more prevalent in the ASD group as compared with controls [odds ratio (OR) 1.75, 95% confidence interval (CI) 1.29–2.38]. This association remained robust after adjusting for birth weight, parental age, parental ethnicity, and maternal immigration [adjusted OR (aOR) 1.61, 95% CI 1.14–2.29]. The most prevalent CM types among the ASD group were circulatory system (2.1 vs. 1.2% among controls) and urogenital organs (1.8 vs. 0.8%). The association between ASD and genital CM was limited to males and persisted in the adjusted models (aOR 2.24, 95% CI 1.16–4.34). In the stratified by sex analysis, a strong association between all non-genitourinary CM and ASD was found in females (aOR 3.47, 95% CI 1.13–10.65). In conclusion, CM, most notably genitourinary in males exclusively, and others (mostly circulatory) in females, are more prevalent in newborns later diagnosed with ASD, as compared with age- and sex-matched controls. These sex-specific CM might represent useful pre- and postnatal markers of ASD, and their presence in newborns at-risk of ASD might indicate earlier and more frequent neurodevelopmental assessments. Our findings might also guide future research of plausible genetic, epigenetic, and prenatal underpinnings of ASD.

## Introduction

The prevalence of autism spectrum disorder (ASD) is on the rise worldwide [1–5]. Progress has been made in recent years in extending worldwide estimates of ASD to previously underrepresented regions of the world, suggesting that as of 2022 the global median prevalence of ASD is 100 per 10,000 individuals (range: 1.09–436.0/10,000) [5]. In the United States, prevalence is 206 per 10,000 individuals, with a median age at diagnosis of 49 months [6]. A large variety of conditions have been reported to be associated with increased risk for ASD diagnosis, but no specific etiology for this disorder has been identified, a fact which most probably points to its multifactorial pathogenesis. More than 60 perinatal and neonatal factors have been identified so far as risk factors for later occurrence of ASD [7]. The increased rate of ASD detection among siblings [8], in addition to higher ASD detection rates among children diagnosed with a wide variety of genetic disorders [9], including various syndromes such as tuberous sclerosis complex, fragile X syndrome, and SHANK3 deficiency [10–13], might imply a probable genetic background for ASD, although no single genomic locus has yet been identified. Other perinatal associations with subsequent ASD diagnosis include advanced paternal age [14–16], maternal age [14], preterm and post-term birth [17–19], low birth weight, offspring of mothers born abroad [17], maternal gestational diabetes, maternal bleeding during pregnancy, and maternal psychoactive medication [20, 21], among others. Specifically, early pregnancy exposure to well-known teratogens (i.e., valproic acid, ethanol, thalidomide, and misoprostol) has been recognized as associated with several types of CM as well as ASD, possibly through similar mechanisms influencing embryogenesis [22]. As might be expected, extensive research has focused on the possible association between ASD and congenital neurological malformations, especially structural brain malformations such as agenesis of the corpus callosum [23] and cerebellar and posterior fossa malformations [24, 25]. Such structural anomalies might provide important information on the etiology and likelihood of later ASD diagnosis. As structural brain anomalies might represent a worrying sign for a systemic embryogenetic disorder, other systemic malformations might also be found at an increased rate among individuals with ASD. Indeed, one study found an association between ASD and congenital diaphragmatic hernia [26], and in one comprehensive environmental study, higher ASD prevalence was linked to congenital malformations of the reproductive system in males only [27], a finding that was replicated in a birth cohort of male singletons in Israel [28].

Based on a robust body of evidence that early intervention can improve prognosis and functioning in individuals with ASD [29–33], the importance of early diagnosis is paramount. Thus, there is a need for pertinent empirical data to guide ASD risk evaluation, by means of identifying all available risk factors and conditions which might represent “red flags” for subsequent ASD diagnosis and indicate early detection [34]. We set out to evaluate the association between CM and ASD in general, and to explore possible associations of ASD with specific malformations. Our aim was to identify CM that are detectable either prenatally or at the time of birth, and that are associated with an increased likelihood of ASD, and which thus might be practically used as early markers for ASD. Given the uncertainty about the influence of all the above-mentioned risk factors in shaping clinical expression in individuals with genetic vulnerability for ASD [35], there is a need for better understanding of the developmental pathways of this highly prevalent disorder.

## Materials and methods

### Study population

Our data were derived from the Israel National Birth Registry (INBR) and the Autism Registry of the Israeli Ministry of Social Affairs (MoSA). The INBR contains complete data on all live births in Israel and on CM. The MoSA registry contains data on the majority of individuals diagnosed with ASD, as the diagnostic information is derived from government-maintained service/benefits registries that record contacts with individuals receiving services and/or benefits for autism [36]. MoSA’s diagnostic procedure for eligibility for services was revised in 2008. Diagnosis by two independent clinicians is now required for all children applying between the ages of 0 and 7 years, which might affect the diagnosis of certain individuals who were born after 2001 and who applied in 2008 or later.

The Perinatal Registry in the Israeli Ministry of Health maintains a dedicated Congenital Malformations Registry. All CM detected at birth are reported to the registry by law within 40 days after birth. Our analysis included all the CM that were reported to the registry. CM were assessed and classified by type and organ system according to the *International Classification of Diseases, Ninth Revision* (ICD-9) and the *International Classification of Diseases, Tenth Revision* (ICD-10). ASD was assessed and diagnosed using the *Diagnostic and Statistical Manual of Mental Disorders-IV Text Revision* (DSM-IV) criteria.

Our study includes data on 4302 singleton pregnancies resulting in live births between January 1, 1993, and early 2006. Twin and triplet pregnancies, totaling 183 cases and 79 control, were excluded from the analysis. Cases of ASD according to the MoSA registry were matched 1:1 on date of birth (non-cases with consecutive ID numbers) and sex. The study population was derived from a national birth cohort, and as such represents the ethnic distribution of the general population of Israel during the years of the cohort.

CM were assessed and classified by type and organ system according to the *International Classification of Diseases, Ninth Revision* (ICD-9) and the *International Classification of Diseases, Tenth Revision* (ICD-10) codes. ASD was assessed and diagnosed using the *Diagnostic and Statistical Manual of Mental Disorders-IV Text Revision* (DSM-IV) criteria.

This study followed the standards and ethics of the American Association for Public Opinion Research reporting guidelines [37] and the Strengthening the Reporting of Observational Studies in Epidemiology (STROBE) reporting guidelines [38].

## Statistical analysis

We conducted a case-control study nested within a cohort of all registered individuals with ASD and an equal number of age- and sex-matched controls, derived from a 14-year (1993–2006) birth cohort. ID number is given to all Israeli newborns immediately after birth. Cases and controls were closely matched on sex and date of birth. Controls were selected based on the next consecutive ID number and the assigned sex at birth in the cohort, skipping siblings. In most instances this resulted in same dates of birth for cases and their controls.

Unadjusted conditional logistic regression with sex and date of birth as strata was performed to determine the crude odds ratio (ORs) and associated 95% confidence intervals (CIs) between any CM and ASD. Multivariable conditional logistic regression was performed to quantify the association between any type of CM and ASD after adjusting for the presence of potential confounders such as birth weight, maternal age, and paternal age (Model 1). Next, ethnicity and maternal immigration were added to the regression model (Model 2). Similarly, we performed the same unadjusted and adjusted conditional logistic regression stratified for sex, considering differences in embryonal pathways between males and females.

**Fig. 1:**
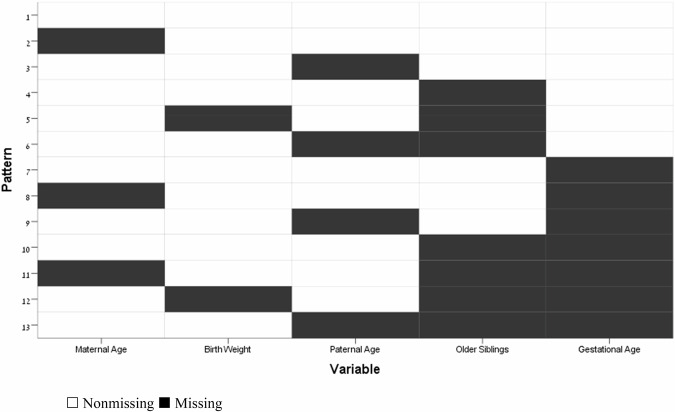
Missing Value Pattern.

For adjusted conditional logistic regressions that utilized birth weight and parental ages, we first analyzed the observed missing data pattern in the entire paired cohort, which was described as *arbitrarily missing* [39] (Fig. 1 in the appendix). We then followed by use of multiple imputation via Markov Chain Monte Carlo (MCMC) methods [40] to impute missing birth weight (0.5%), maternal age at birth (0.2%), and paternal age at birth (5.6%). We generated 20 imputed data sets on which we ran our analysis, and then combined the results by taking the average of the regression coefficients across data sets using Rubin and Schenker’s formula [41] to estimate the variance. In a sensitivity analysis designed to assess the robustness of our data imputation strategy, we utilized MCMC methods to further generate 20 imputed datasets in which the equations were modeled, under an assumption that missing birth data belong in the year of outcome assessment (a 4-year delay from actual birth) or end of follow-up, whichever came first. Parental missing value parameters were mirrored to belong to parents of the opposite sex, providing children whose paternal or maternal data are missing with a ‘parental same-sex data donor’. This was achieved by transplanting instances of paternal missing values in the maternal variables and vice versa, while also accounting for variability in parental age differences in the cohort. All data analysis and statistics programming were conducted using IBM SPSS Statistics version 29.0.2.

## Results

Basic demographic characteristics of the study and control groups in the final study population are shown in Table 1. Because of frequency matching on sex, approximately 82% of both cases and controls were male.

**Table 1:** Characteristics of Cases with Autism Spectrum Disorder and Controls.

Characteristic	Cases^a^ (n = 2099)		Controls^a^ (n = 2203)		Crude OR (95% CI)
	N	%	N	%	
Sex					
Male	1732	82.5	1806	82.0	
Female	365	17.4	369	18.0	
Maternal immigration	530	25.3	381	17.3	
Paternal immigration	488	23.2	381	17.3	
Maternal age [years, means (SD)]	30.68 (5.29)		29.09 (5.56)		
Paternal age [years, means (SD)]	33.83 (5.80)		32.45 (6.15)		
Older siblings [count, means (SD)]^b^	2.03 (1.34)		2.82 (2.11)		
Ethnicity					
Jewish	1921	91.5	1506	68.4	Reference
Arab	73	3.5	577	26.2	1.03 (0.69, 1.54)
Druze	7	0.3	50	2.3	0.10 (0.06, 0.16)
Non-Jewish, NA	29	1.4	14	0.6	0.12 (0.05, 0.33)
Unknown	69	3.3	56	2.5	1.73 (0.78, 3.83)
Birth year					
1993	100	4.8	102	4.6	
1994	81	3.9	84	3.8	
1995	106	5.1	110	5.0	
1996	124	5.9	129	5.9	
1997	149	7.1	152	6.9	
1998	163	7.8	173	7.9	
1999	160	7.6	174	7.9	
2000	157	7.5	163	7.4	
2001	188	9.0	191	8.7	
2002	224	10.7	234	10.6	
2003	250	11.9	272	12.3	
2004	222	10.6	234	10.6	
2005	155	7.4	164	7.4	
2006	20	1.0	21	1.0	
Birth weight [grams, means (SD)]^b^	3261.10 (585.18)		3291.39 (516.10)		
Gestational age [weeks, means (SD)]^b^	38.86 (2.42)		39.24 (1.99)		
ICD-10 codes					
No Congenital malformations	1974	94.0	2130	96.7	Reference
Any congenital malformation	125	6.0	73	3.3	1.75 (1.29, 2.38)
Q10–18: Malformations of eye, ear, face and neck	3	0.1	3	0.1	0.67 (0.11, 3.99)
Q20–28: Malformations of the circulatory system	45	2.1	27	1.2	1.74 (1.04, 2.91)
Q38–45: Malformations of the digestive system	5	0.2	1	0.0	4.00 (0.45, 35.79)
Q50–56: Congenital malformations of genital organs	38	1.8	18	0.8	2.31 (1.29, 4.16)
Q60–64: Malformations of the urinary system	17	0.8	8	0.4	2.00 (0.86, 4.67)
Q65–79: Malformations and deformations of the musculoskeletal system	15	0.7	8	0.4	1.63 (0.67, 3.92)
Q80–89: Other congenital malformations	6	0.3	9	0.4	0.86 (0.29, 2.55)
Q90–99: Chromosomal abnormalities, not elsewhere classified	4	0.2	1	0.0	4.00 (0.45, 35.79)

^a^Singletons only. A total of 183 cases and 79 controls who are non-singletons (twins and triplets) are not shown.

^b^Data on maternal age were available for n = 4293, and data on paternal age were available for 4060 children. Gestational age data were available for n = 2475, and birth weight data were available for n = 4279. Data on siblings were available for n = 3237.

The relative frequency per study groups of different types of CM, classified by organ systems, is presented in Table 1. CM were identified in 198 individuals: 125 (6%) of the ASD cases and 73 (3.3%) of the controls. The most common malformation types in the study group included: circulatory system (2.1% in the cases vs 1.2% in the controls), genital organs (1.8 vs 0.8%), and urinary system (0.8 vs 0.4%). The prevalence of all other malformations, except for ophthalmic, ear, face, and neck malformations, was also higher in diagnosed cases compared with controls.

To evaluate the association between specific CM and ASD, we calculated crude and adjusted ORs (Table 2). Adjusted models did not adjust for gestational age, which was missing data substantially (44.8% of cases). The likelihood of all types of congenital malformations was higher in the study group compared with controls (OR 1.75, 95% CI 1.29–2.38). This association remained robust when adjusted for birth weight and parental ages (aOR 1.78, 95% CI 1.30–2.43). The association was slightly weakened in the model that also adjusted for parental ethnicity and maternal immigration (aOR 1.61, 95% CI 1.14–2.29). A statistically significant increased likelihood of circulatory system malformations was found among ASD cases in the unadjusted model (OR 1.74, 95% CI 1.04–2.91) and in model 1 (aOR 1.71, 95% CI 1.01–2.89), but this association was attenuated after also adjusting for ethnicity and maternal immigration (aOR 1.62, 95% 0.89–2.95). By contrast, the association of congenital malformations of the genital organs with ASD, found to be statistically significant in the unadjusted model (OR 2.31, 95% CI 1.29–4.16), persisted after adjusting for all potential confounders. When observing earlier periods (2000-born or earlier) vs. later periods (2001-born and beyond) of the cohort, no particular effect of temporality is apparent except the above-mentioned associations not consistently crossing the significance threshold (Table 3).

**Table 2:** Crude and Adjusted Odds Ratios for the Association between Autism Spectrum Disorder and Congenital Malformations.

Congenital Malformation	Cases^a^ (n = 2099)		Controls^a^ (n = 2203)		Crude OR		OR adjusted for birth weight and parental age^b^		OR adjusted for birth weight, parental age^b^, nationality and maternal immigration	
ICD-10 codes	N	%	N	%	OR	95% CI	OR	95% CI	OR	95% CI
None	1974	94.0	2130	96.7	1.00	Reference	1.00	Reference	1.00	Reference
Any congenital malformation	125	6.0	73	3.3	1.75^*^	1.29, 2.38	1.78^*^	1.30, 2.43	1.61^*^	1.14, 2.29
Q10–18: Malformations of eye, ear, face and neck	3	0.1	3	0.1	0.67	0.11, 3.99	0.66	0.11, 4.06	0.70	0.11, 4.29
Q20–28: Malformations of the circulatory system	45	2.1	27	1.2	1.74^*^	1.04, 2.91	1.71^*^	1.01, 2.89	1.62	0.89, 2.95
Q38–45: Malformations of the digestive system	5	0.2	1	0.0	4.00	0.45, 35.79	4.86	0.52, 44.95	3.37	0.36, 32.02
Q50–56: Congenital malformations of genital organs	38	1.8	18	0.8	2.31^*^	1.29, 4.16	2.51^*^	1.38, 4.55	2.21^*^	1.14, 4.30
Q60–64: Malformations of the urinary system	17	0.8	8	0.4	2.00	0.86, 4.67	2.16	0.91, 5.16	1.64	0.61, 4.37
Q65–79: Malformations and deformations of the musculoskeletal system	15	0.7	8	0.4	1.63	0.67, 3.92	1.50	0.61, 3.68	1.17	0.43, 3.17
Q80–89: Other congenital malformations	6	0.3	9	0.4	0.86	0.29, 2.55	0.96	0.31, 2.91	1.66	0.45, 6.16
Q90–99: Chromosomal abnormalities, not elsewhere classified	4	0.2	1	0.0	4.00	0.45, 35.79	3.25	0.35, 30.13	3.06	0.31, 30.34

^*^p < 0.05

^a^Singletons only. A total of 183 cases and 79 controls who are non-singletons (twins and triplets) are not shown.

^b^Data on birth weight were available for n = 4279. Maternal age data were available for n = 4293, and paternal age data were available for n = 4060.

**Table 3:** Time Period Comparison of the Crude and Adjusted Odds Ratios for the Association between Autism Spectrum Disorder and Congenital Malformations.

Time period	Congenital Malformation	Cases^a^		Controls^a^		Crude OR		OR adjusted for birth weight and parental age^b^	
1993–2000	ICD-10 codes	N	%	N	%	OR	95% CI	OR	95% CI
	None	981	94.3	1056	97.1	1.00	Reference	1.00	Reference
	Any congenital malformation	59	5.7	31	2.9	1.89*	1.19, 3.01	1.84^*^	1.14, 2.96
	Q10–18: Malformations of eye, ear, face and neck	3	0.3	1	0.1	2.00	0.18, 22.06	2.28	0.20, 25.79
	Q20–28: Malformations of the circulatory system	15	1.4	7	0.6	2.00	0.75, 5.33	1.84	0.68, 4.99
	Q38–45: Malformations of the digestive system	4	0.4	1	0.1	3.00	0.31, 28.84	3.22	0.32, 31.97
	Q50–56: Congenital malformations of genital organs	20	1.9	11	1.0	2.00	0.94, 4.27	2.06	0.95, 4.47
	Q60–64: Malformations of the urinary system	4	0.4	3	0.3	1.00	0.20, 4.96	0.96	0.18, 5.03
	Q65–79: Malformations and deformations of the musculoskeletal system	8	0.8	4	0.4	1.50	0.42, 5.32	1.40	0.39, 5.06
	Q80–89: Other congenital malformations	5	0.5	4	0.4	2.50	0.49, 12.89	2.64	0.50, 13.78
	Q90–99: Chromosomal abnormalities, not elsewhere classified	3	0.3	1	0.1	3.00	0.31, 28.84	2.33	0.23, 23.32
2001–2006	ICD-10 codes	N	%	N	%	OR	95% CI	OR	95% CI
	None	993	93.8	1074	96.2	1.00	Reference	1.00	Reference
	Any congenital malformation	66	6.2	42	3.8	1.66^*^	1.11, 2.48	1.75^*^	1.16, 2.65
	Q10–18: Malformations of eye, ear, face and neck	0	0.0	2	0.2		p = 0.57		p = 0.95
	Q20–28: Malformations of the circulatory system	30	2.8	20	1.8	1.65	0.90, 3.01	1.67	0.90, 3.11
	Q38–45: Malformations of the digestive system	1	0.1	0	0.0		p = 0.61		p = 0.97
	Q50–56: Congenital malformations of genital organs	18	1.7	7	0.6	2.83^*^	1.12, 7.19	3.31^*^	1.29, 8.51
	Q60–64: Malformations of the urinary system	13	1.2	5	0.4	2.60	0.93, 7.29	2.99*	1.04, 8.60
	Q65–79: Malformations and deformations of the musculoskeletal system	7	0.7	4	0.4	1.75	0.51, 5.98	1.61	0.46, 5.69
	Q80–89: Other congenital malformations	1	0.1	5	0.4	0.20	0.02, 1.71	0.23	0.03, 2.04
	Q90–99: Chromosomal abnormalities, not elsewhere classified	1	0.1	0	0.0		p = 0.61		p = 0.97

^*^p < 0.05

^a^Singletons only. A total of 183 cases and 79 controls who are non-singletons (twins and triplets) are not shown.

^b^Data on birth weight were available for n = 4279. Maternal age data were available for n = 4293, and paternal age data were available for n = 4060.

In the stratified-by-sex analysis, the association between ASD and any CM was statistically significant in females (OR = 2.83) and in males (OR = 1.64) (Table 4). This association remained significant in all adjusted models only in males (unadjusted: OR 1.64, 95% CI 1.19–2.27; model 1: aOR 1.75, 95% CI 1.26–2.44; model 2: aOR 1.64, 95% CI 1.13–2.37). The association between ASD diagnosis and congenital malformations of genital organs was also found only in males and remained statistically significant in the adjusted models (unadjusted model: OR = 2.31, 95% CI 1.29–4.16; model 1: aOR 2.54, 95% CI 1.40–4.63; model 2: aOR = 2.24, 95% CI 1.16–4.34). The association between urinary system malformations and ASD was statistically significant only in male offspring in model 2 (aOR 2.70, 95% CI 1.01–7.18), presumably due to cells with small numbers: 17 out of all malformation types (0.8%) in the study group vs 8 (0.4%) in the controls.

**Table 4:** Sex-Stratified Odds Ratios for the Association between Autism Spectrum Disorder and Congenital Malformations.

Sex	Congenital Malformation	Cases^a^ (n = 2097)		Controls^a^ (n = 2202)		Crude OR		OR adjusted for birth weight and parental age^b^		OR adjusted for birth weight, parental age^b^, nationality and maternal immigration	
		N	%	N	%	OR	95% CI	OR	95% CI	OR	95% CI
Male	None	1629	94.1	1740	96.3	1.00	Reference	1.00	Reference	1.00	Reference
	Any congenital malformation	103	5.9	66	3.7	1.64^*^	1.19, 2.27	1.75^*^	1.26, 2.44	1.64^*^	1.13, 2.37
	Congenital urogenital malformations	53	3.1	24	1.3	2.32^*^	1.41, 3.82	2.59^*^	1.55, 4.32	2.21^*^	1.25, 3.92
	Congenital malformations of genital organs	38	2.2	18	1.0	2.31^*^	1.29, 4.16	2.54^*^	1.40, 4.63	2.24^*^	1.16, 4.34
	Malformations of the urinary system	15	0.9	6	0.3	2.33	0.90, 6.07	2.70^*^	1.01, 7.18	2.12	0.69, 6.53
	All other congenital malformations	54	3.1	44	2.4	1.28	0.84, 1.95	1.34	0.87, 2.06	1.33	0.82, 2.15
	Malformations of eye, ear, face and neck	1	0.1	3	0.2	0.33	0.35, 3.21	0.32	0.03, 3.11	0.35	0.04, 3.46
	Malformations of the circulatory system	33	1.9	24	1.3	1.48	0.85, 2.57	1.57	0.89, 2.78	1.60	0.84, 3.05
	Malformations of the digestive system	5	0.3	0	0.0		p = 0.31		p = 0.93		p = 0.93
	Malformations and deformations of the musculoskeletal system	11	0.6	8	0.4	1.25	0.49, 3.17	1.15	0.45, 2.97	0.87	0.30, 2.49
	Other congenital malformations	5	0.3	8	0.4	0.83	0.25, 2.73	1.06	0.32, 3.55	2.10	0.51, 8.61
	Chromosomal abnormalities, not elsewhere classified	2	0.1	1	0.1	2.00	0.18, 22.06	1.93	0.17, 22.05	2.16	0.18, 25.91
Female	None	343	94.0	389	98.2	1.00	Reference	1.00	Reference	1.00	Reference
	Any congenital malformation	22	6.0	7	1.8	2.83^*^	1.12, 7.19	2.42	0.93, 6.28	1.71	0.61, 4.82
	Congenital urogenital malformations	2	0.5	2	0.5	1.00	0.14, 7.10	0.65	0.08, 5.13	0.82	0.10, 6.67
	Congenital malformations of genital organs	0	0.0	0	0.0						
	Malformations of the urinary system	2	0.5	2	0.5	1.00	0.14–7.10	0.65	0.08–5.13	0.82	0.10, 6.67
	All other congenital malformations	21	5.8	5	1.3	4.00^*^	1.34, 11.97	3.47^*^	1.13, 10.65	2.30	0.69, 7.63
	Malformations of eye, ear, face and neck	2	0.5	0	0.0		p = 0.61		p = 0.95		p = 0.95
	Malformations of the circulatory system	12	3.3	3	0.8	4.50	0.97, 20.83	3.86	0.80, 18.55	1.94	0.34, 11.23
	Malformations of the digestive system	0	0.0	1	0.3		p = 0.61		p = 0.98		p = 0.97
	Malformations and deformations of the musculoskeletal system	7	1.8	0	0.0		p = 0.38		p = 0.94		p = 0.94
	Other congenital malformations	1	0.3	1	0.3	1.00	0.06, 15.99	0.82	0.05, 13.48	0.94	0.06, 15.57
	Chromosomal abnormalities, not elsewhere classified	2	0.5	0	0.0		p = 0.47		p = 0.96		p = 0.95

^*^p < 0.05

^a^Singletons only. A total of 183 cases and 79 controls who are non-singletons (twins and triplets) are not shown. Undetermined sex not shown. Individuals with multiple malformations are accurately represented in each respective cell, hence total numbers represent individuals and not malformations.

^b^Data on birth weight were available for n = 4279. Maternal age data were available for n = 4293, and paternal age data were available for n = 4060.

Two findings emerged as related to female sex. First, when examining all congenital malformation other than the types that were found to have association with male sex (i.e., genital organs, urinary systems), we found a strong association with female sex (crude OR 4.00, 95% CI 1.34–11.97), also when adjusted to birth weight and maternal and paternal ages (aOR 3.47, 95% CI 1.13–10.65). Second, there was a statistically nonsignificant trend for association between circulatory congenital malformations and ASD in females, in both crude and adjusted models (respectively: OR 4.50, model 1 aOR 3.86, model 2 aOR 1.94).

Missingness in the adjusted models was not likely to affect the robustness of the estimates of those effects according to the results of the sensitivity analysis (Table 5).

**Table 5:** Sensitivity Analysis^a^ of the Association between Autism Spectrum Disorder and Congenital Malformations.

Sex	Congenital Malformation	OR adjusted for birth weight and parental age^b^		OR adjusted for birth weight, parental age^b^, nationality and maternal immigration	
		OR	95% CI	OR	95% CI
Male +	None	1.00	Reference	1.00	Reference
Female	Any congenital malformation	1.78*	1.30, 2.43	1.61*	1.13, 2.28
	Malformations of eye, ear, face and neck	0.66	0.11, 4.08	0.70	0.12, 4.33
	Malformations of the circulatory system	1.71*	1.01, 2.89	1.62	0.89, 2.95
	Malformations of the digestive system	4.85	0.52, 44.97	3.16	0.33, 29.80
	Congenital malformations of genital organs	2.51*	1.38, 4.55	2.21*	1.14, 4.30
	Malformations of the urinary system	2.15	0.90, 5.14	1.62	0.61, 4.31
	Malformations and deformations of the musculoskeletal system	1.51	0.62, 3.70	1.17	0.43, 3.20
	Other congenital malformations	0.96	0.31, 2.91	1.67	0.45, 6.19
	Chromosomal abnormalities, not elsewhere classified	3.26	0.35, 30.17	2.96	0.30, 29.31
Male	None	1.00	Reference	1.00	Reference
	Any congenital malformation	1.75*	1.25, 2.44	1.63*	1.12, 2.36
	Congenital urogenital malformations	2.59*	1.55, 4.32	2.21*	1.25, 3.91
	Congenital malformations of genital organs	2.54*	1.40, 4.63	2.24*	1.16, 4.34
	Malformations of the urinary system	2.69*	1.01, 7.17	2.10	0.68, 6.45
	All other congenital malformations	1.34	0.87, 2.06	1.32	0.81, 2.13
	Malformations of eye, ear, face and neck	0.32	0.03, 3.12	0.35	0.04, 3.48
	Malformations of the circulatory system	1.57	0.88, 2.78	1.59	0.84, 3.03
	Malformations of the digestive system		p = 0.93		p = 0.93
	Malformations and deformations of the musculoskeletal system	1.16	0.45, 2.98	0.87	0.30, 2.50
	Other congenital malformations	1.06	0.32, 3.54	2.11	0.52, 8.62
	Chromosomal abnormalities, not elsewhere classified	1.95	0.17, 22.24	2.12	0.18, 25.36
Female	None	1.00	Reference	1.00	Reference
	Any congenital malformation	2.46	0.95, 6.39	1.72	0.61, 4.84
	Congenital urogenital malformations	0.66	0.08, 5.17	0.82	0.10, 6.65
	Congenital malformations of genital organs				
	Malformations of the urinary system	0.66	0.08, 5.17	0.82	0.10, 6.65
	All other congenital malformations	3.55*	1.16, 10.89	2.32	0.70, 7.68
	Malformations of eye, ear, face and neck		p = 0.95		p = 0.95
	Malformations of the circulatory system	4.00	0.84, 19.16	2.05	0.36, 11.75
	Malformations of the digestive system		p = 0.98		p = 0.97
	Malformations and deformations of the musculoskeletal system		p = 0.94		p = 0.94
	Other congenital malformations	0.84	0.05, 13.75	0.95	0.06, 15.57
	Chromosomal abnormalities, not elsewhere classified		p = 0.96		p = 0.95

^*^p < 0.05

^a^All models utilized a multiple imputation procedure in which birth data generation was 4-year delayed (roughly to the year of outcome assessment) and completed with ‘parental same-sex data donor’.

^b^Data on birth weight were missing for 0.5% of children. Maternal age data were missing for 0.2% of children, and paternal age data were missing for 5.6% of children.

## Discussion

The overall objective of our study was to identify CM which might flag an increased likelihood of ASD pre- or neonatally. We found a higher overall prevalence of CM of all types among individuals with ASD as compared with age- and sex-matched newborns without ASD, as well as in comparison with the general Israeli population and global prevalence statistics [42–44]. While previous studies have described an increased prevalence of specific CM in subjects diagnosed with ASD, to the best of our knowledge, our study is the first to report an association between ASD and *all* congenital malformations.

When the results are stratified by sex, we found that this association was predominantly driven by urogenital malformations in males and circulatory malformations in females. A similar association between congenital urogenital malformations in male offspring and ASD was described in a male-only cohort in Israel [28]. Those findings remained consistent also when adjusted for paternal and maternal age. However, it is worth noting that the detection of urogenital malformations tends to be more straightforward in male infants when compared to females. In certain instances, these malformations may only be diagnosed during puberty among females. Consequently, further data is required to accurately assess this disparity.

All other, non-urogenital congenital malformations (consisting mostly of circulatory system congenital malformations) were statistically significantly associated with ASD in females only, and data suggest a potential association between congenital malformations of the circulatory system and ASD may be more prominent in females. A similar association between congenital heart disease and later ASD was previously reported in a large case-control study, which did not explore the role of newborns’ sex in said association [45]. In our study, probably due to the limited number of cases, this association did not reach the significance threshold, and further study is needed in order to examine this matter more closely.

The differential association of CM types and ASD between the sexes might imply a differential impact of sex chromosomes, as well as serum androgen levels, on developmental and embryonal pathways that are involved in ASD pathomechanism. A long standing stipulation that the hemizygous nature of the X chromosome in males is central to their “male vulnerability” [46], has laid ground to research into associations between X-linked genetic alterations and neurodevelopmental ailments [47]. Similarly, it has been proposed that the absence of Y chromosome, or the presence of two X chromosomes may provide the fetus with elevated protection against development of ASD [48]. The current study supports that abnormal neurological development may be chromosomally moderated by sex chromosomes, while maintaining that there may be several pathways underlying ASD which are potentially differentiated by sex.

A recent meta-analysis confirmed the robust association between elevated androgen levels during multiple developmental stages and ASD [49], seemingly extending support to the extreme male brain (EMB) theory, which postulates a unidirectional relationship between the development of sex-differentiated behavior and ASD [50]. Hypothetically, one might expect individuals with subnormal intrauterine androgen levels to be less susceptible ASD. The observed association between congenital malformations of the male reproductive system, thought to involve deficiencies in prenatal androgen signaling [51–53], and ASD poses a threat to the above theoretical reasoning. Our findings are consistent with a similar study focused on male newborns only and found the association to persist even when controlling for chromosomal and congenital anomalies affecting the nervous system [28]. Deviations from normal intrauterine androgen exposure is known to affect female gametes as well, potentially leading to ovarian dysfunction [54], which is not diagnosed at the neonatal stage.

ASD is viewed as an umbrella term for diverse phenotypes with discrete origins versus a phenomenon with a similar genetic or hormonal developmental mechanism for both males and females. There is evidence that excess androgen at the embryonic stage may be deleterious to the development of the circulatory system and cardiac function [55, 56]. In the current study an association between all non-genitourinary congenital malformations and ASD is observed among females only, and we posit that this association is driven mostly by congenital malformations of the circulatory system. Since we note that androgen deficiency is thought to underlie the CM we found to be associated with male ASD and, on the flip side, excess in androgen may lead to CM more observable in female ASD, it is worthwhile to further explore how gonadal steroid hormones contribute to ASD pathogenesis and the potentially inverse ways in which they act in males and females.

The main strength of our study is derived from our large, population-based cohort, which enables a real-life representation of neurotypical and ASD- diagnosed population living in Israel. All citizens in Israel are covered by a universal health insurance which diminishes the likelihood of selection bias due to socioeconomic status. Additionally, the use of two independent sources of registered data and their comparison confirms the blindness of the doctors who diagnose each ailment, therefore minimizing information bias or selection bias.

Our study has several limitations. First, our data extraction stopped in 2006, possibly reflecting passed times and changing approaches to diagnosis and treatment. However, the significant improvements in prenatal ultrasound which enable early diagnosis of major structural malformations has triggered a rise in cases resulting in termination of pregnancies [57, 58], and cancelling the possibility of analyzing ASD development during early life among newborns with serious CM. Second, lower ASD diagnosis rates in the past might more accurately reflect a trend for changes in diagnostic criteria, which have led to an increase in the observed prevalence [59, 60], and might imply that overdiagnosis of this condition might exist nowadays. A statistical limitation inherent to an inspection of the full range of ICD-9 and ICD-10 CM diagnoses is that some, such as “Chromosomal abnormalities, not elsewhere specified” are rarely indicated and challenge this study’s inferential capacity to confirm or disprove an association between ASD and the rarest of CM. Third, survival bias in which cohort members with congenital malformations might have died before diagnosed with ASD as compared with cohort members without CM. Fourth, the potential underreporting of ASD. Assuming the association between ASD and CM is similar among Jews and non-Jews, and considering the fact that ASD is underreported in the non-Jewish population in Israel, we think that this will lead to underestimation of the strength of the observed association. Fifth, presumably most of the cohort was past the mean age of ASD diagnosis at the end of follow up. It is possible, however, that some of those born between 2004–2006 were misclassified as controls due to shorter length of follow up. Based on the association found in the earlier years of birth, such misclassification might have biased the results towards the null. Sixth, data on gestational age are missing in over 40% of the cohort. The missing gestational age data are most likely missing not at random, and therefore dependent on unobserved or unknown factors. When such missing not at random is present, statistical adjustment for the missing information becomes highly unreliable [61]. Seventh, our study was conducted only among the Israeli population, with its immigrant profile of wide genetic variability limiting the generalizability of our findings. Information regarding genetics or family history of ASD were unavailable.

## Conclusion

In conclusion, in this large population-based Israeli nested case-control study we found that CM, most notably genitourinary in males exclusively, and others (mostly circulatory) in females, are more prevalent in newborns later diagnosed with ASD, as compared with age- and sex-matched controls. Confounding by sex is implausible because of the matched design. These sex-specific CM might represent useful pre- and postnatal markers of ASD, and their presence in newborns at-risk of ASD might indicate earlier and more frequent neurodevelopmental assessments. Our findings might also guide future research of plausible genetic, epigenetic, and prenatal underpinnings of ASD.

## Data Availability

The datasets analyzed during the current study were obtained from the internal Israel National Birth Registry and the Autism Registry of the Israeli Ministry of Social Affairs and are not publicly available due to data protection and confidentiality restrictions. De-identified data may be made available from the corresponding author upon reasonable request and subject to institutional approval.
